# Supplementary material for: Reduced Etch Lag and High Aspect Ratios by Deep Reactive Ion Etching (DRIE)
Source: Micromachines (Basel). 2021 May 10;12(5):542. doi: 10.3390/mi12050542 (PMC8150727; doi:10.3390/mi12050542)
Supplement: Supplementary file 1 [file micromachines-12-00542-s001.zip › micromachines-1202243-supplementary.pdf]

Supporting Information for "Reduced etch lag and high aspect ratios by deep reactive ion etching (DRIE)"

Michael S. Gerlt,* Nino F. Läubli, Michel Manser, Bradley J. Nelson, and Jürg Dual

E-mail: gerlt@imes.mavt.ethz.ch

Supporting Figures

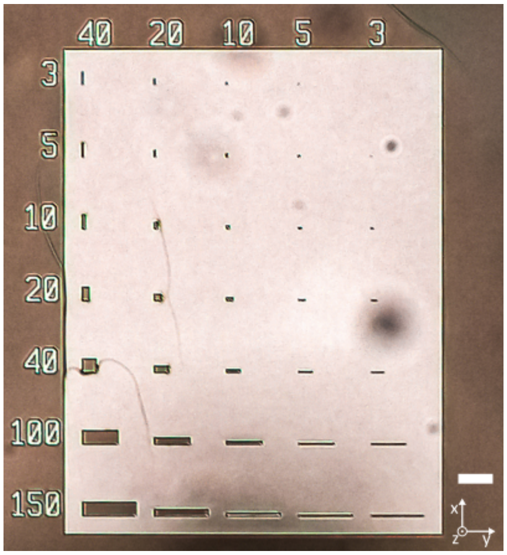

Figure S1: **Quality analysis of the photolithography process.** Optical microscopy image of S1813 photoresist after development (1.4 µm thickness). The numbers on the top and the side indicate the width (x-direction) and the length (y-direction) of the structures, respectively. All features could be reproduced with a sufficient accuracy. Scale bar corresponding to 100 µm.

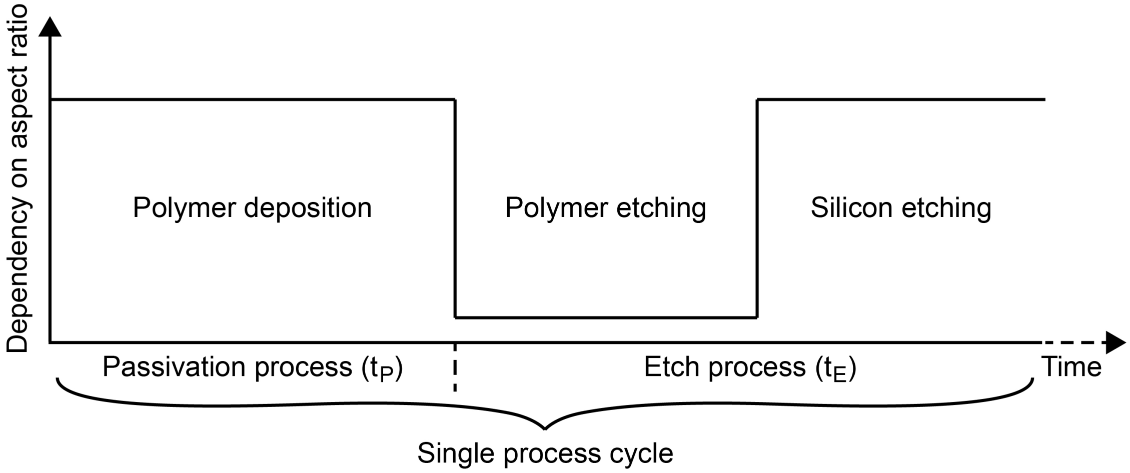

Figure S2: **Bosch process cycle.** The graph shows a single cycle of the Bosch process consisting of a passivation and an etching step. The etching procedure can be further divided into a mostly physical polymer etching and a purely chemical silicon etching. For each step, its dependency on the structures aspect ratio is provided qualitatively, with physical steps having a low dependence while chemical processes have a high dependence due to the limited availability of reactive species in confined areas.

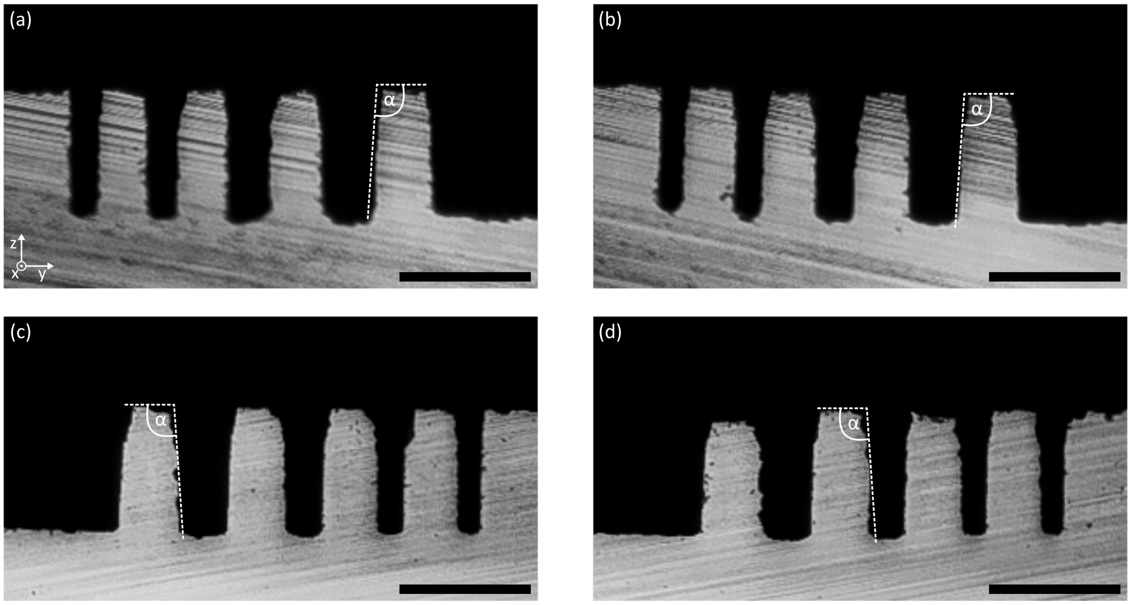

Figure S3: **Reproducibility analysis of the optimised two-step Bosch process.** We measured two etching profiles on two wafers. The etch angles α were measured at three different locations and are (a) 92.2°, (b) 90.9°, (c) 90.6°, and (d) 90.9°. In average, the etch angle was $91.1° \pm 0.6°$. Scale bars corresponding to 100 µm

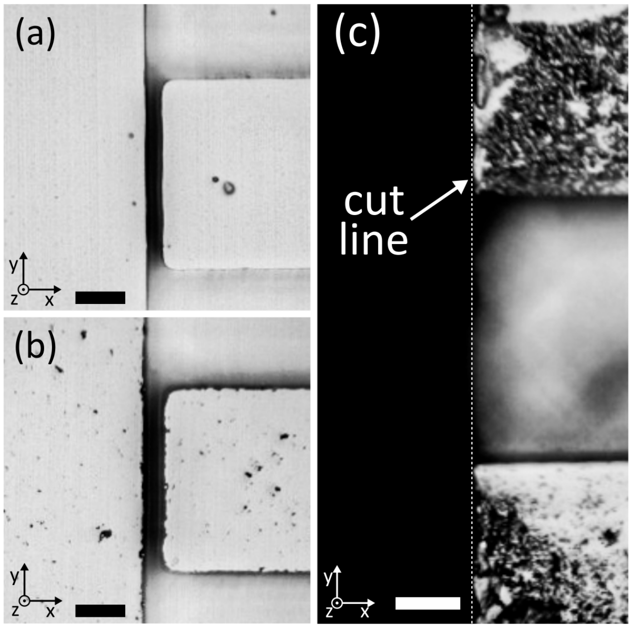

Figure S4: **Influence of dicing on the sidewall roughness.** Optical microscopy images of a 20 µm wide trench on a wafer that (a) was not diced and (b) was diced. The dicing process induced vibrations that influenced the sidewall roughness, even though the cut was performed at a different location. (c) optical microscopy image of a cut line. the surface roughness induced by the cut process is clearly visible. Scale bars corresponding to 40 µm

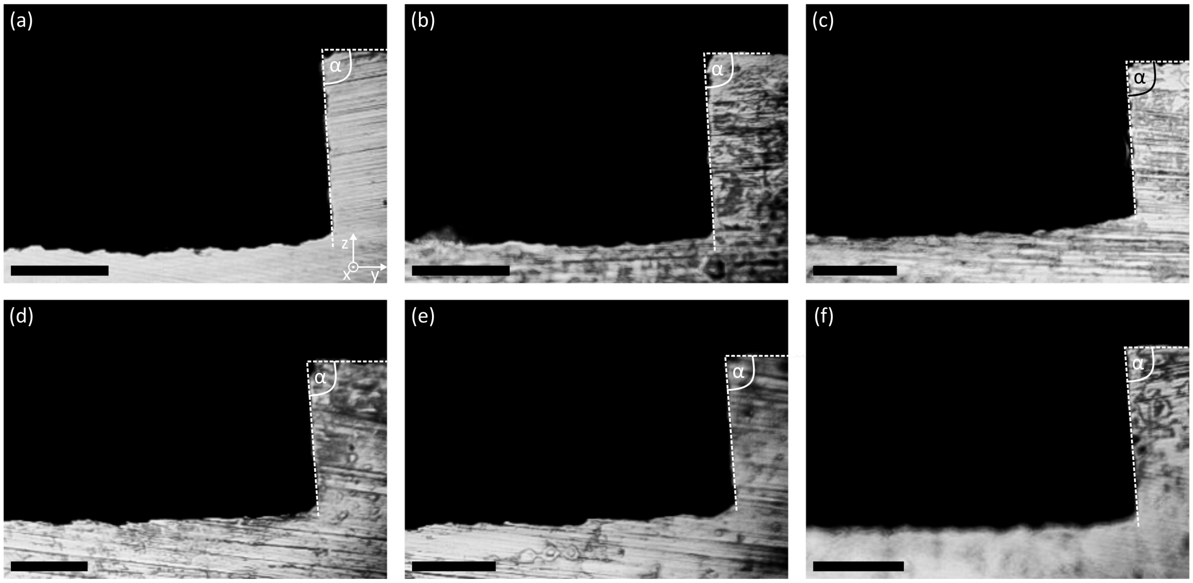

Figure S5: **Reproducibility analysis of the standard three-step Bosch process.** We measured six different channel geometries. The etch angles α were measured at three different locations and are (a) 86.0°, (b) 85.4°, (c) 86.6°, (d) 85.9°, (e) 86.3°, and (f) 85.7°. In average, the etch angle was 86.0° ± 0.4°. Scale bars corresponding to 100 µm

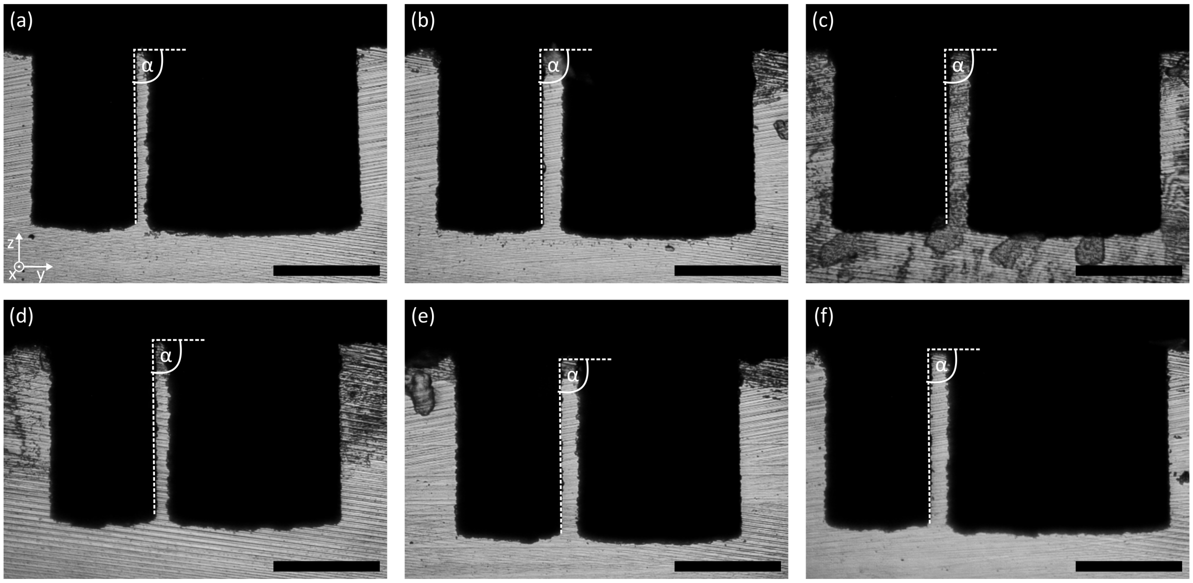

Figure S6: **Reproducibility analysis of the optimised three-step Bosch process.** We measured six different channel geometries. The etch angles α were measured at three different locations and are (a) 89.4°, (b) 89.4°, (c) 89.6°, (d) 89.6°, (e) 89.8°, and (f) 89.7°. In average, the etch angle was 89.6° ± 0.1°. Scale bars corresponding to 100 µm

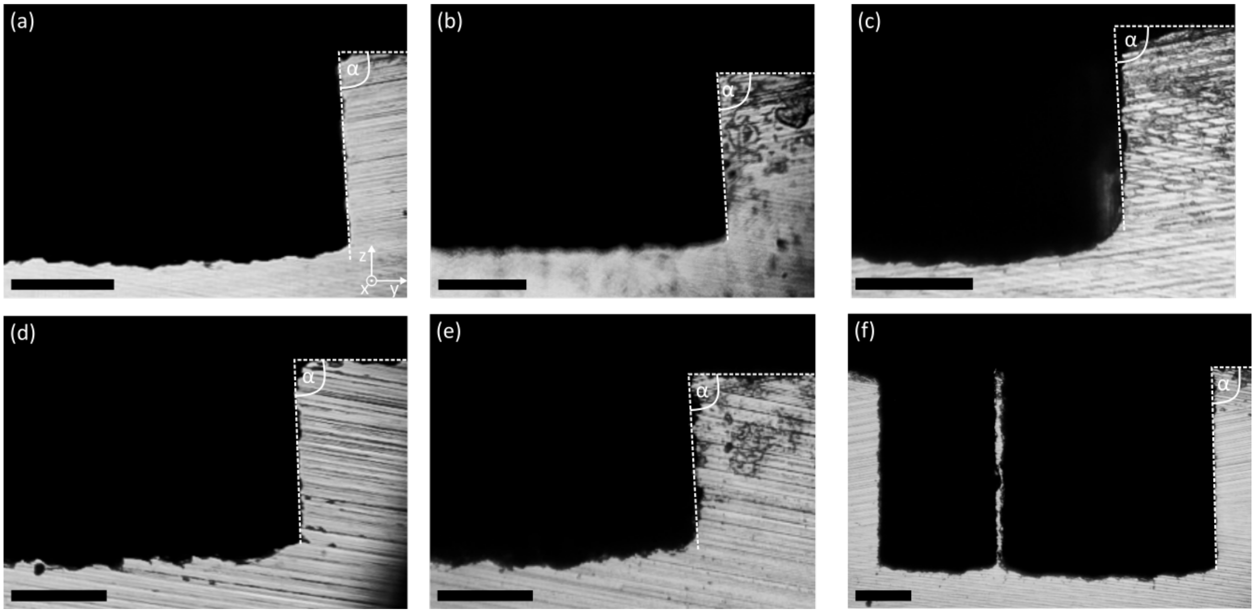

Figure S7: **Three-step Bosch process development.** We tested the influence of various parameters on the etch angle and selectivity. The corresponding process parameters can be found in Supporting Table S3. (a) corresponds to the standard three-Step Bosch process (Supporting Table S4) and (f) corresponds to the optimised three-Step Bosch process (Supporting Table S5)

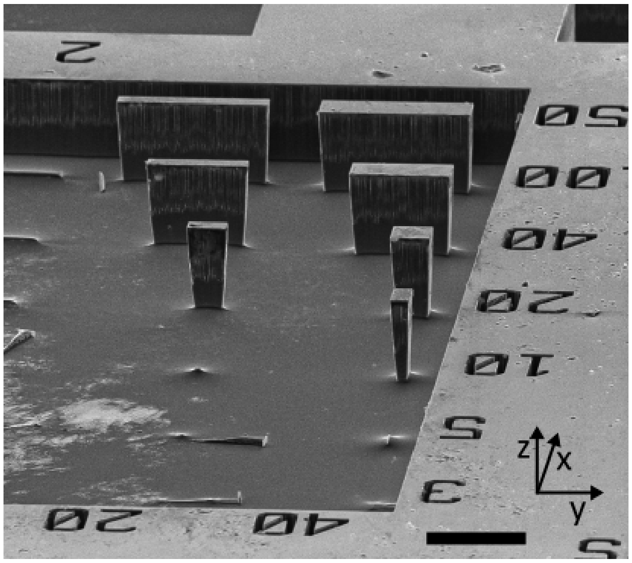

Figure S8: **Scanning electron microscopy image of the standard three-step Bosch process.** The tilt of the SEM head allowed us to inspect the structures stability. Scale bar corresponding to 100 µm

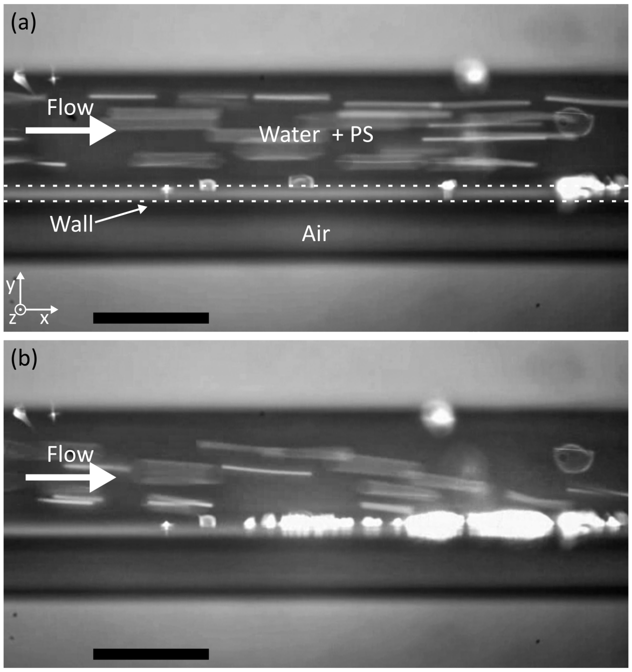

Figure S9: **Impermeability of the thin wall.** Microscope pictures of the device shown in the top of Figure 3 (c). The Microfluidic channels were sealed with glass and through one of the channels, water with 0.005 % w/v fluorescent polystyrene particles (5 µm diameter) was flown with a speed of $7.5\,\mathrm{mm\,s^{-1}}$. (a) Even though the wall in between the two channels is only 6 µm thick, no water is leaking into the lower air-channel, which is apparent by the absence of PS particles. (b) Upon excitation, particles can be trapped at the thin wall, which is beneficial for various biomicrofluidic applications. Scale bars corresponding to 200 µm.

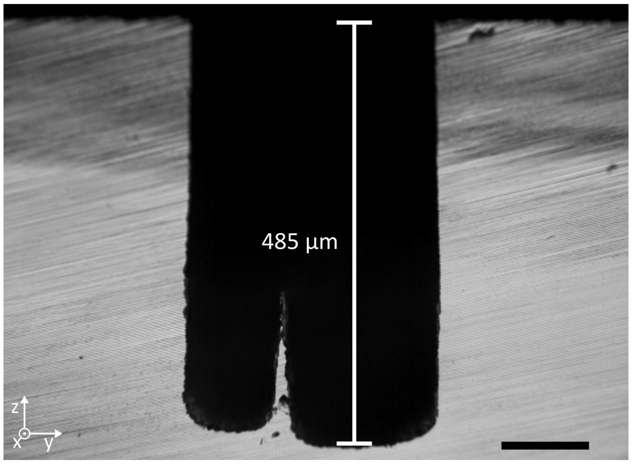

Figure S10: **Deep etching.** With a 1.3 µm thin resist layer, we etched 100 µm (left) and 200 µm (right) wide channels 485 µm deep into a silicon wafer, corresponding to a selectivity of over 350. The 8 µm thin wall in between the two channels could not be maintained due to the etch angle. Scale bar corresponding to 100 µm.

Supporting Tables

Two-step Bosch Process

Table S1: Process parameters of the standard two-step Bosch process (Fig. 2(a) top)

Step Name	Step Time [ms]	Pressure [mTorr]	ICP Power [W]	HF Power [W]	C_4F_8 Flow [sccm]	SF_6 Flow [sccm]
DEP	3000	50	1500	0	200	10
ETCH	6000	25	1500	100	10	100

Table S2: Process parameters of our optimised two-step Bosch process (Fig. 2(a) bottom). Changes regarding the standard process are highlighted.

Step Name	Step Time [ms]	Pressure [mTorr]	ICP Power [W]	HF Power [W]	C_4F_8 Flow [sccm]	SF_6 Flow [sccm]
DEP	6000	70	1500	0	200	10
ETCH	6000	32	1400	100	10	100

Three-step Bosch Process

Table S3: Table of the three step Bosch process incremental improvements. dep, etch1 and etch2 are the three steps of the process (see Figure 2 (a)). p_e is the pressure of the last sub-step during etch1, ICP is the ICP power during etch2, rate is the etch rate of the silicon, sel. is the selectivity, and e.a. is the etch angle. For the processes with red. gas flow, the gas flow of all gases during etch2 was reduced by 50 %

	dep	etch1		etch2						
Process	time [s]	time [s]	p_e [mTorr]	time [s]	ICP [W]	Mask material	rate [µm/s]	sel.	e.a. [°]	Comments
Fig. S10 (a)	0.75	0.6	25.0	2.3	3500	SiO_2	0.20	90	86.3	
Fig. S10 (b)	0.78	0.7	23.3	1.9	2500	SiO_2	0.25	900	87	
Fig. S10 (c)	1.15	0.6	20.0	2.2	2000	PR	0.13	152	89.1	red. gas flow
Fig. S10 (d)	1.35	0.7	20.0	1.9	2000	PR	0.12	108	89.4	red. gas flow
Fig. S10 (e)	1.55	0.8	20.0	1.6	2000	PR	0.09	103	89.7	red. gas flow
Fig. S10 (f)	1.35	0.7	20.0	1.9	2500	PR	0.16	350	89.6	

Table S4: Process parameters of the standard three-step Bosch process (Fig. 3(a) top)

Step Name	Step Time [ms]	Pressure [mTorr]	ICP Power [W]	HF Power [W]	C_4F_8 Flow [sccm]	SF_6 Flow [sccm]
DEP STAGE1	400	120	2500	0	280	10
DEP1B	200	120	2500	0	10	200
DEP1C	100	0	2000	0	10	200
ETCH1	300	25	2000	75	10	200
ETCH2	2300	120	3500	0	10	800
ETCH2C	350	120	2500	0	280	10

Table S5: Process parameters of our optimised three-step Bosch process (Fig. 3(a) bottom). Changes regarding the standard process are highlighted.

Step Name	Step Time [ms]	Pressure [mTorr]	ICP Power [W]	HF Power [W]	C_4F_8 Flow [sccm]	SF_6 Flow [sccm]
DEP STAGE1	1000	120	2500	0	280	10
DEP1B	200	120	2500	0	10	200
DEP1C	100	0	2000	0	10	200
ETCH1	400	20	2000	75	10	200
ETCH2	1900	120	2500	0	10	800
ETCH2C	350	120	2500	0	280	10
